# IL-21 and anti-CD40 restore Bcl-2 family protein imbalance in vitro in low-survival CD27^+^ B cells from CVID patients

**DOI:** 10.1038/s41419-018-1191-8

**Published:** 2018-11-21

**Authors:** Antonio López-Gómez, Antonio Clemente, Vanesa Cunill, Jaime Pons, Joana M. Ferrer

**Affiliations:** 1 Immunology Department, Son Espases Hospital, Palma, Balearic Islands Spain; 2 Human Immunopathology Research Laboratory, Institut d’Investigació Sanitària de les Illes Balears, Palma, Spain; 3 Clinical Trials and Methodology Support Platform, Institut d’Investigació Sanitària de les Illes Balears, Palma, Spain

## Abstract

Common variable immunodeficiency (CVID) is characterized by an abnormal B cell differentiation to memory and antibody-secreting B cells. The defective functionality of CVID patients’ B cells could be the consequence of alterations in apoptosis regulation. We studied the balance of Bcl-2 family anti-/pro-apoptotic proteins to identify molecular mechanisms that could underlie B cell survival defects in CVID. We used flow cytometry to investigate Bcl-2, Bcl-XL, Bax, and Bim expression in B cells ex vivo and after anti-CD40 or anti-BCR activation with or without IL-21, besides to spontaneous and stimulation-induced Caspase-3 activation and viable/apoptotic B cell subpopulations. We found increased basal levels of Bax and Bim in CVID B cells that correlated with low viability and high Caspase-3 activation only in CD27^+^ B cells, particularly in a subgroup of apoptosis-prone CVID (AP-CVID) patients with low peripheral B cell counts and high autoimmunity prevalence (mostly cytopenias). We detected a broad B cell defect in CVID regarding Bcl-2 and Bcl-XL induction, irrespective of the stimulus used. Therefore, peripheral CVID memory B cells are prompted to die from apoptosis due to a constitutive Bcl-2 family protein imbalance and defective protection from activation-induced apoptosis. Interestingly, anti-CD40 and IL-21 induced normal and even higher levels of Bcl-XL, respectively, in CD27^+^ B cells from AP-CVID, which was accompanied by cell viability increase. Thus low-survival memory B cells from AP-CVID can overcome their cell death regulation defects through pro-survival signals provided by T cells. In conclusion, we identify apoptosis regulation defects as disease-contributing factors in CVID. B cell counts and case history of cytopenias might be useful to predict positive responses to therapeutic approaches targeting T-dependent signaling pathways.

## Introduction

Common variable immunodeficiency (CVID) is the commonest symptomatic primary humoral immunodeficiency, characterized by hypogammaglobulinemia and poor response to vaccination. CVID patients not only suffer from respiratory and/or gut recurrent infections but also additional non-infectious features, including autoimmune and autoinflammatory processes or lymphoproliferative disorders. Patients benefit from substitutive gammaglobulin therapy^1–3^. Although many immunological defects have been described in CVID, pathogenesis of the disease remains unknown^4^. An abnormal late B cell differentiation to memory B cells and antibody-secreting cells (ASC) is a consistent CVID finding. Accordingly, patients have been classified depending on naive, non-switched and switched memory B cell numbers^5–7^.

The generation of memory B cells and ASC is crucial to establish humoral immune responses. T cell cooperation is essential and occurs through contact between T cell membrane molecules and corresponding B cell ligands^8^, whose relevance has been exemplified by naturally occurring immunodeficiencies^9^. Secretion of cytokines like interleukin (IL)-21, mainly produced by activated follicular T cells, also instructs B cell differentiation^10–13^. Apart from their effect on proliferation and differentiation, these stimuli also influence apoptosis/survival balance needed to preserve B cell homeostasis, which shows specific requirements depending on B cell maturation and activation status. Activation threshold required for B cell differentiation is significantly lower while apoptosis susceptibility is higher in memory compared to naive B cells^14,15^. IL-21 co-stimulation is essential in human B cell differentiation to ASC, but T cell interaction is mandatory^16^. Thus B cell receptor (BCR) activation induces B cell apoptosis, even enhanced by IL-21, if survival signals provided through CD40 contact are absent. Accordingly, the stimulatory/inhibitory effect of IL-21 depends on the accompanying signal and the B cell subpopulation evaluated^17,18^. We previously demonstrated that memory B cell loss in a CVID patients’ subgroup (with compromised memory B cell compartment) could be the consequence of increased susceptibility to activation-induced apoptosis^15^. Moreover, several studies reported that CVID subgroups can be distinguished depending on B cell functionality in vitro^19,20^ that may be consequence of different apoptosis regulation outcomes.

Programmed cell death is a widespread pathway whose regulation deeply influences hematopoietic system^21^. Intrinsic/mitochondrial apoptosis is triggered by internal stimuli, as excessive DNA damage, and extrinsic apoptosis by external stimuli through tumor necrosis factor (TNF) receptor superfamily. Anti-apoptotic Bcl-2 family proteins, mostly Bcl-2, Bcl-XL, or MCL-1, are crucial in mitochondrial apoptosis prevention. Pro-apoptotic Bcl-2 family members classify as initiators (Bim, Bid, or Bad among others) or effectors (Bax and Bak)^21,22^. These signals distinctively influence the survival/apoptosis balance depending on the cell type, and when cell death threshold is exceeded, Caspase activation cascade drives intracellular proteolytic degradation^23^. Immature and mature naive B cell survival is differently promoted by Bcl-XL and Bcl-2, respectively, in mice^24^. However, mechanisms involved in prolonged memory B cell survival and the influence of different stimuli on peripheral B cell survival through Bcl-2 family members remain obscure in humans.

The aim of our study was to determine whether alterations in anti-/pro-apoptotic Bcl-2 family protein modulation could influence the imbalanced homeostasis and defective CVID B cell functionality contributing to immunodeficiency. We hypothesize that these alterations could shape heterogeneous CVID B cell functionality and/or result in distinct clinical phenotypes that could benefit from personalized therapeutic approaches targeting cell survival mechanisms.

## Materials and methods

### Patients

Twenty CVID patients were selected according to diagnostic criteria of the International Union for Immunological Societies scientific group for primary immunodeficiency diseases. Patients received intravenous gammaglobulin therapy every 21–28 days and did not suffer from infections at the time of the study. Most patients (*n* = 16) did not receive corticosteroids, immunosuppressive drugs and/or biologic therapies at least 3 months prior to the time of study. Patients 1 and 7 were treated with low doses of prednisone, patient 13 was treated with tacrolimus and patient 3 was treated with low doses of prednisone, tacrolimus and mycophenolate. Peripheral blood samples were collected before immunoglobulin (Ig) replacement. Supplementary Table 1 summarizes the patients’ age, gender, Igs serum levels at diagnosis, absolute counts of CD4^+^ T cells, percentages and absolute counts of B cell subpopulations, EUROclass group^7^, autoimmune manifestations, presence of enteropathy, lymphoproliferative complications, and malignancies. Age- and sex-matched healthy blood donors were included as controls. The study was conducted according to the ethical guidelines of the 1975 Declaration of Helsinki and approved by CEIC (Balearic Islands Clinical Research Ethics Committee; IB 2517/15). Informed consent was obtained from all subjects.

### Cell culture

Peripheral blood mononuclear cells (PBMCs) isolated from heparinized blood by density gradient centrifugation were re-suspended in RPMI-1640 medium supplemented with 10% heat inactivated fetal calf serum and antibiotics (penicillin-streptomycin). In all, 1–4 × 10^6^ cells were cultured in 24-well plates and stimulated with F(ab)_2_ goat anti-human IgA+IgG+IgM (5 μg/mL; Jackson ImmunoResearch) or with anti-human CD40/TNFRSF5 antibody (1 μg/mL; R&D Systems) in the presence or absence of human recombinant IL-21 (100 ng/mL; Biosource). Cultures were maintained at 37 °C in a 5% CO_2_ atmosphere during 20 h.

### Flow cytometry

#### Intracellular staining of Bcl-2, Bcl-XL, Bax, and Bim

An intracellular staining protocol was performed to evaluate Bcl-2, Bcl-XL, Bax, and Bim expression by flow cytometry in B cells following the manufacturer’s instructions (IntraPrep Permeabilization Reagent from Beckman Coulter). Ex vivo basal expression was detected in B cells from freshly isolated PBMCs and in vitro stimulation-induced expression was evaluated in B cells from cultured PBMCs.

Briefly, 1 × 10^6^ freshly isolated or cultured cells were stained for 30 min with Fixable Viability Dye-eFluor520 (eBioscience) at 4 °C, washed, and resuspended in 100 μL cold phosphate-buffered saline (PBS). Cells were stained 15 min with anti-CD19-PCy7 and anti-CD27-PCy5 (both from Beckman Coulter). After surface staining, cells were fixed with formaldehyde solution for 15 min, washed with cold PBS, and permeabilized with saponin solution 20 min at room temperature (RT; 25 °C) in the dark. Intracellular staining was performed with mouse anti-human-Bcl-2-PE (clone Bcl-2/100, BD Pharmingen), mouse anti-human-Bcl-XL-PE (clone 7B2.5, Abcam), mouse anti-human-Bax-PE (clone 2D2, Santa Cruz Biotechnology), or rabbit anti-human-Bim-PE (clone C34C5, Cell Signaling) monoclonal antibodies, added within the last 15 min of the permeabilization step. Finally, cells were washed, resuspended in 400 μL of PBS, and analyzed.

**Fig. 1:**
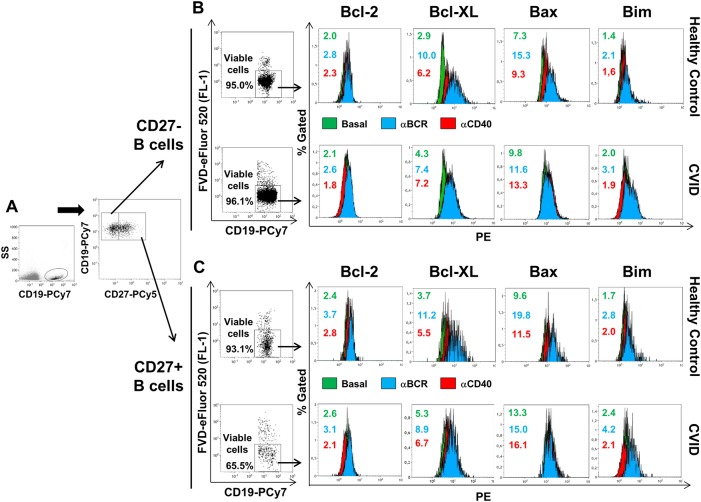
Gating strategy to evaluate Bcl-2 family protein expression in healthy controls and CVID B cells. Total naive CD19^+^CD27^–^ and memory CD19^+^CD27^+^ B cell subpopulations were gated (**a**) and death cells (Viability Dye-eFluor 520 positive) were discarded (dot plots in **b**, **c**) previously to evaluate Bcl-2, Bcl-XL, Bax, and Bim expression in viable naive CD19^+^CD27^–^ (histograms in **b**) and memory CD19^+^CD27^+^ (histograms in **c**) B cells. Histograms and numbers represent Bcl-2, Bcl-XL, Bax, and Bim MFI levels from a representative healthy control (upper rows in **b**, **c**) and CVID patient (lower rows in **b**, **c**) in unstimulated B cells (green) or after stimulation with anti-BCR (blue) or anti-CD40 (red). MFI median fluorescence intensity

Total Bcl-2, Bcl-XL, Bax, and Bim median fluorescence intensity (MFI) was measured in previously gated naive (CD19^+^CD27^–^) and memory (CD19^+^CD27^+^) viable (Viability Dye-eFluor520^–^) B cells (Fig. 1). MFI increase/decrease induced by each single stimulus or their combinations related to baseline was expressed as a ratio: [post-stimulation MFI/baseline MFI].

#### Apoptosis evaluation by Caspase-3 activation analysis

Active Caspase-3, Annexin-V, and 7-aminoactinomycin D (7-AAD)-Viability Dye staining protocol was performed to evaluate the apoptosis of B cells from PBMCs cultures, following the manufacturer's instructions (CaspGLOW™ Fluorescein Active Caspase Staining Kit from Invitrogen).

Briefly, 1 × 10^6^ cultured cells were stained for 15 min with anti-CD19-APC and anti-CD27-PCy7 (both from Beckman Coulter). After surface staining, cells were resuspended in 300 μL of supplemented RPMI-1640 medium and 1 μL of FITC-Val-Ala-Asp Fluoromethyl Ketone (VAD-FMK that irreversibly binds to the active Caspase-3 enzyme) was added. Cells were incubated at 37 °C in a 5% CO_2_ atmosphere during 1 h, washed twice, and resuspended in 100 μL of Annexin-V binding buffer (Invitrogen). Next, 5 µL of Annexin-V-PE (Invitrogen) and 5 µL of 7-AAD-Viability Dye (Beckman Coulter) were added, cells were incubated 15 min at RT (25 °C) in the dark and immediately analyzed after addition of 300 μL of Annexin-V binding buffer.

**Fig. 2:**
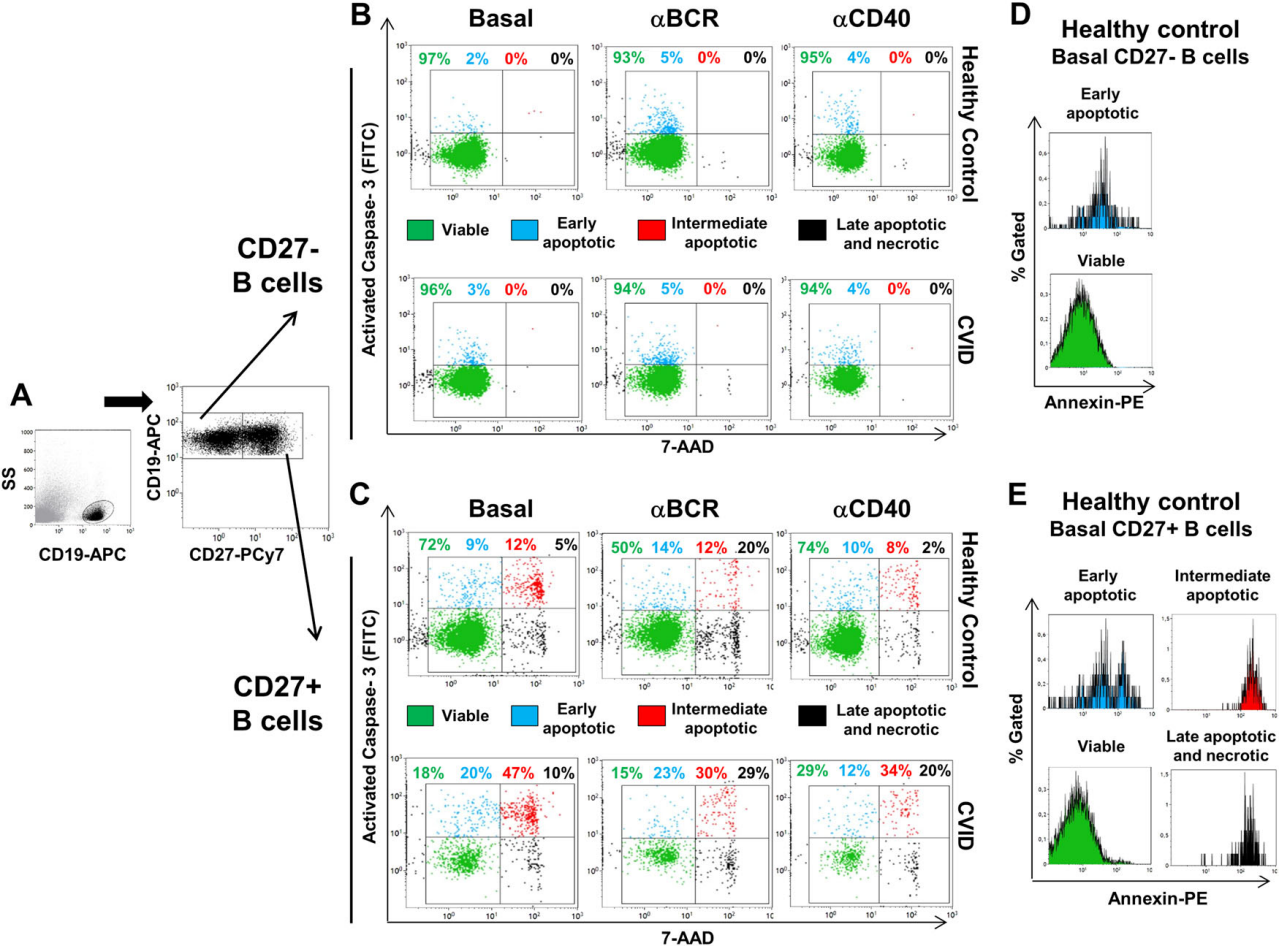
Caspase-3 activation analysis and evaluation of distinct stages of apoptosis in healthy controls and CVID B cells. Total naive CD19^+^CD27^–^ and memory CD19^+^CD27^+^ B cell subpopulations were gated (**a**) and levels of apoptotic cells were evaluated in a three-colour staining and gating strategy. Dot plots and percentages represent viable active-Caspase-3^–^/7-AAD^–^ (green dots in lower left quadrants), early-apoptotic active-Caspase-3^+^/7-AAD^–^ (blue dots in upper left quadrants), intermediate-apoptotic active-Caspase-3^+^/7-AAD^+^ (red dots in upper right quadrants), and late-apoptotic/necrotic active-Caspase-3^–^/7-AAD^+^ (black dots in lower right quadrants) naive CD19^+^CD27^–^ and memory CD19^+^CD27^+^ B cells (**b**, **c**, respectively) from a representative healthy control (upper rows) and CVID patient (lower rows) in unstimulated cells or after stimulation with anti-BCR or anti-CD40. Histograms represent Annexin-V expression in CD19^+^CD27^–^ (**d**) and CD19^+^CD27^+^ (**e**) unstimulated B cells from a representative healthy control and were used to confirm distinct stages of apoptosis in B cell subpopulations

Analysis of four major populations of viable and apoptotic cells was performed by flow cytometry as previously described^25^. Percentage of viable (active-Caspase-3^–^/Annexin-V^–^/7-AAD^–^), early-apoptotic (active-Caspase-3^+^/Annexin-V^+^/7-AAD^–^), intermediate-apoptotic (active-Caspase-3^+^/Annexin-V^+^/7-AAD^+^), late-apoptotic/necrotic (active-Caspase-3^–^/Annexin-V^+^/7-AAD^+^) cells, and total percentage of Caspase-3-activated (active-Caspase-3^+^) cells were measured in previously gated naive (CD19^+^CD27^–^) and memory (CD19^+^CD27^+^) B cells (Fig. 2). This multi-parameter analysis of apoptosis provides more accurate information than single-parameter assays that give an ambiguous result of apoptotic cell frequency. Total Caspase-3-activated cell percentage increase/decrease or viable and apoptotic cell percentage increase/decrease induced by each single stimulus or their combinations related to baseline was expressed as a ratio: [post-stimulation %/baseline %].

### Statistical analysis

Statistical analysis was performed using the GraphPad Prism software (San Diego, CA). Data are expressed as median and percentiles 25th and 75th. Mann–Whitney test was used to compare differences between CD27^–^ and CD27^+^ B cell subpopulations and differences between total CVID patients group and controls. Kruskal–Wallis test was used to compare differences between CVID patients’ subgroups and controls. Wilcoxon test was used to compare differences between two paired groups of treatments (basal and post-stimulation conditions or each single stimulus with or without IL-21). Correlation between variables was measured using Spearman’s correlation coefficient. Fisher's exact test was used in the analysis of contingency tables. A *P* value <0.05 was considered statistically significant.

## Results

### CVID CD27^–^ and CD27^+^ B cells exhibit higher constitutive expression of Bax and Bim

We evaluated anti-apoptotic Bcl-2 and Bcl-XL or pro-apoptotic Bax and Bim basal levels in naive (CD27^–^) and memory (CD27^+^) viable B cells (Fig. 1). Bcl-2, Bcl-XL, Bax, and Bim basal levels were greater in CD27^+^ than in CD27^–^ control B cells both ex vivo and in vitro (Supplementary Figure 1, panels A and B, respectively).

**Fig. 3:**
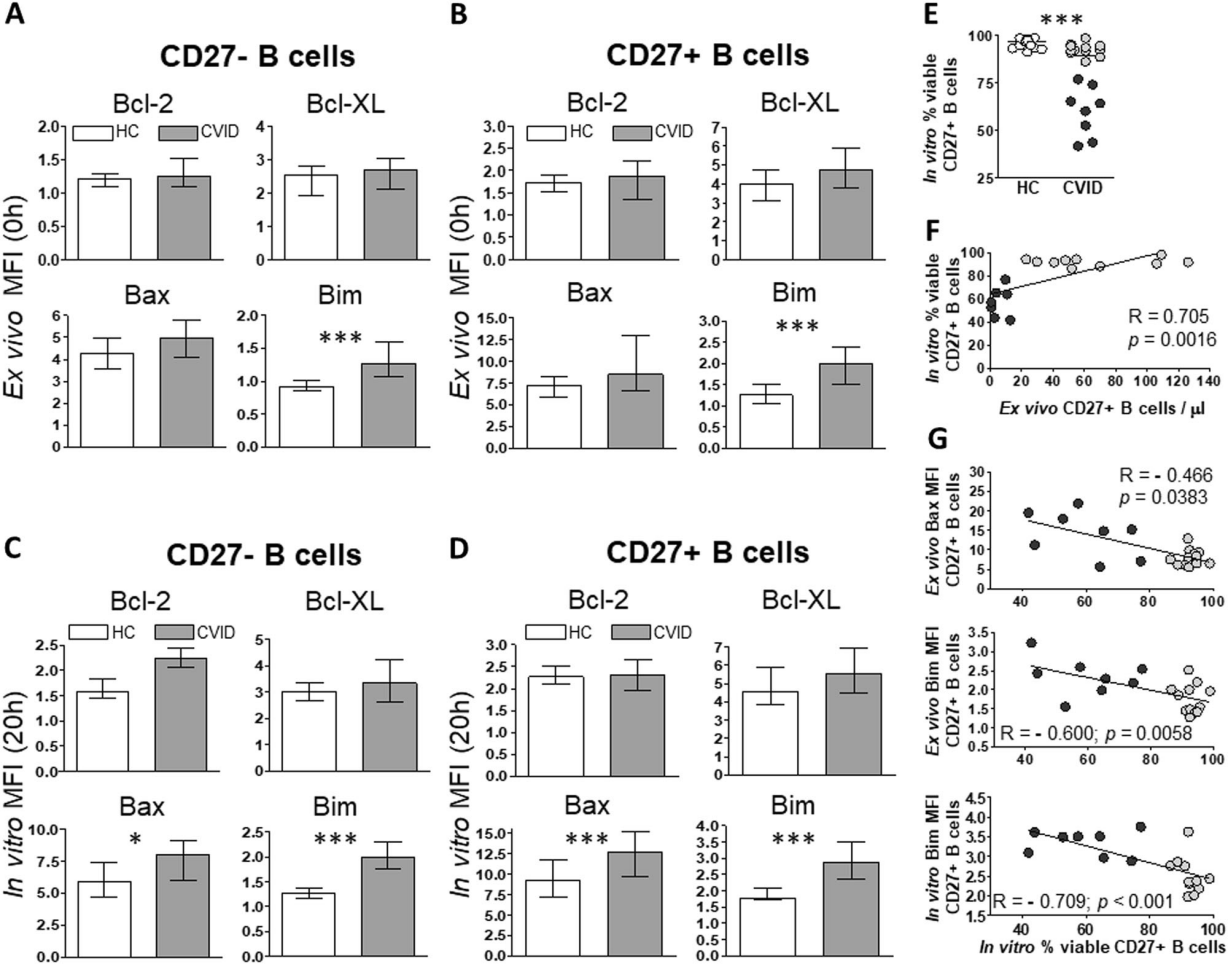
Increased ex vivo and in vitro basal levels of Bax and Bim in CVID B cells. MFI levels of Bcl-2 (upper left), Bcl-XL (upper right), Bax (lower left), and Bim (lower right) in naive CD19^+^CD27^−^ and memory CD19^+^CD27^+^ B cells detected in ex vivo freshly isolated cells (**a**, **b**) and in vitro cultured cells without stimulation (**c**, **d**) from healthy controls (white bars) and CVID patients (gray bars). In vitro percentages of viable (Viability Dye-eFluor520 negative) memory CD19^+^CD27^+^ B cells from healthy controls (white dots), CVID (light gray dots), and apoptosis-prone CVID (dark gray dots) patients (**e**). Data are given as medians and 25-75th percentiles (Mann–Whitney test *P* values: *P < 0.05; ***P < 0.001). Correlation between absolute counts of ex vivo memory CD19^+^CD27^+^ B cells and in vitro percentages of viable memory CD19^+^CD27^+^ unstimulated B cells from CVID patients (**f**). Correlations between in vitro percentages of viable memory CD19^+^CD27^+^ B cells and ex vivo MFI levels of Bax (upper **g**) and Bim (middle **g**) and in vitro MFI levels of Bim (lower **g**) in memory CD19^+^CD27^+^ B cells from CVID patients. MFI median fluorescence intensity, HC healthy controls (*n* = 20), CVID common variable immunodeficiency patients (*n* = 20); *R* Spearman’s correlation coefficient

We found higher ex vivo basal levels of Bim in CD27^–^ and CD27^+^ B cells from CVID patients compared to controls (Fig. 3a, b). In vitro Bax and Bim basal levels were also higher in CVID patients compared to controls both in CD27^–^ and CD27^+^ B cells (Fig. 3c, d). No differences were found between CVID patients and controls when Bcl-2 and Bcl-XL basal levels were compared, neither in CD27^–^ nor in CD27^+^ B cells (Fig. 3a–d).

### Higher levels of Bax and Bim correlate with low viability of CVID CD27^+^ B cells

We had previously described that, if maintained unstimulated, spontaneous apoptosis is higher in CD27^+^ than in CD27^–^ B cells^15^. To evaluate the impact of pro-apoptotic protein expression, we explored correlations between Bax and Bim basal levels and B cell viability in non-stimulated cultures, as well as percentages or absolute counts of peripheral blood CD27^–^ and CD27^+^ B cells.

Percentages of viable CD27^+^ B cells in vitro were significantly lower in patients than in controls (Fig. 3e), specially in 8 patients whose viable CD27^+^ B cell levels were >5 standard deviations below the mean of both controls and remaining patients (dark gray dots in Fig. 3e). There was a positive correlation between absolute counts of peripheral blood CVID CD27^+^ B cells and percentages of viable CVID CD27^+^ B cells in vitro (Fig. 3f). Interestingly, the eight patients with lower percentages of viable CD27^+^ B cells in vitro clustered separately when we correlated peripheral blood absolute counts and in vitro CVID CD27^+^ B cell viability (<20 cells/μL and <80% viable cells, respectively) (dark gray dots in Fig. 3f).

There was a negative correlation between percentages of viable CVID CD27^+^ B cells in vitro and basal MFI levels of Bax and Bim in CVID CD27^+^ B cells. Previously identified eight patients differentiated again from the remaining CVID patient cluster (dark gray dots in Fig. 3g). No correlations were found in CVID CD27^–^ B cells (data not shown). These findings prompted us to separately study these eight patients in further analyses. From now on, we will refer to this group as apoptosis-prone CVID (AP-CVID) patients. Clinical and laboratory characteristics were compared between AP and remaining CVID patients (Supplementary Table 2). We found that 50.0% of AP-CVID patients had peripheral B cell levels below normal value versus 0% of remaining CVID patients (*P* = 0.014, Fisher’s exact test). Moreover, when retrospectively analyzed, these four AP-CVID patients had maintained B cells below normal level the past 5 years. Interestingly, autoimmune manifestations (mostly cytopenias) were detected in 75.0% of AP-CVID versus 16.7% of remaining CVID patients (*P* = 0.019, Fisher’s exact test).

### Increased spontaneous Caspase-3 activation on AP-CVID CD27^+^ B cells

**Fig. 4:**
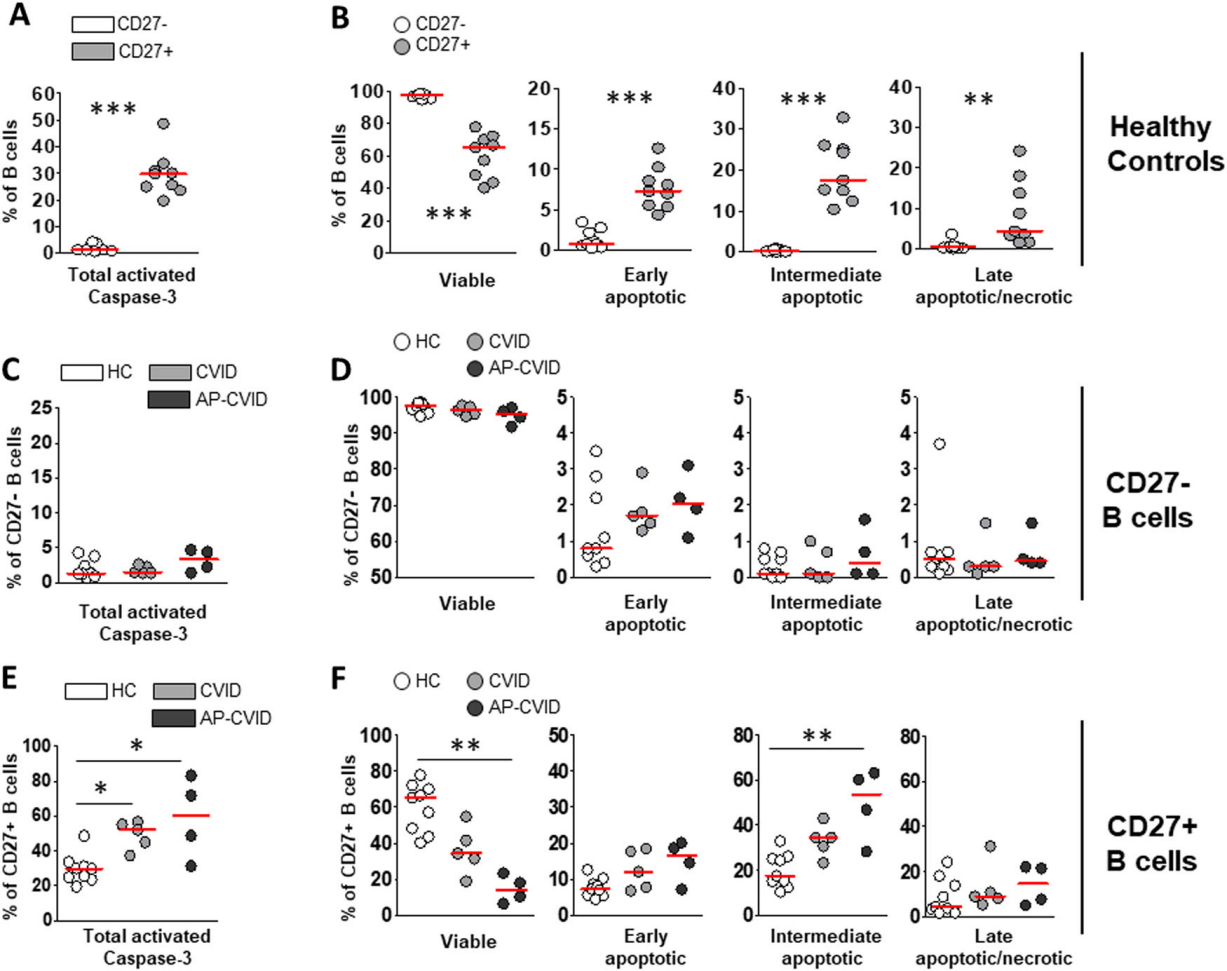
Higher spontaneous Caspase-3 activation and increased apoptotic cell populations in unstimulated CVID memory B cells. Percentages of total Caspase-3-activated naive CD19^+^CD27^−^ (white circles) and memory CD19^+^CD27^+^ (light gray circles) unstimulated B cells from healthy controls (**a**). Percentages of viable, early-apoptotic, intermediate-apoptotic, and late-apoptotic/necrotic cells detected in previously gated naive CD19^+^CD27^–^ (white circles) and memory CD19^+^CD27^+^ (light gray circles) unstimulated B cells from healthy controls (**b**). Percentages of total Caspase-3-activated naive CD19^+^CD27^–^ (**c**) and memory CD19^+^CD27^+^ (**e**) unstimulated B cells from healthy controls (white circles), CVID (light gray circles) and apoptosis-prone CVID (dark gray circles) patients. Percentages of viable, early-apoptotic, intermediate-apoptotic, and late-apoptotic/necrotic cells detected in previously gated naive CD19^+^CD27^–^ (**d**) and memory CD19^+^CD27^+^ (**f**) unstimulated B cells from healthy controls (white circles), CVID (light gray circles), and apoptosis-prone CVID (dark gray circles) patients. Data are given as medians (Mann–Whitney and Kruskal–Wallis test *P* values: *P < 0.05; **P < 0.01; ***P < 0.001). HC healthy controls (*n* = 9), CVID common variable immunodeficiency patients (*n* = 5), AP-CVID apoptosis-prone CVID patients (*n* = 4)

To confirm that Bax and Bim higher basal levels detected in CVID B cells could result in apoptosis-mediated cell death, we evaluated spontaneous Caspase-3 activation (apoptosis trigger final event) in unstimulated cultures (Fig. 2). Higher percentage of total Caspase-3-activated CD27^+^ than CD27^–^ B cells were found in unstimulated cultures from controls (Fig. 4a), in agreement with its lower viability. When distinct stages of apoptosis on CD27^+^ and CD27^–^ control B cells were individually compared, we found lower percentages of viable and higher percentages of early-apoptotic, intermediate-apoptotic, and late-apoptotic/necrotic cells in the CD27^+^ B cell subpopulation. We never detected intermediate-apoptotic or late-apoptotic/necrotic CD27^–^ B cells (Figs. 2 and 4b). These results suggest distinct apoptosis kinetics between both B cell subpopulations.

Percentages of total Caspase-3-activated CD27^–^ B cells were very low both in controls and CVID patients (Fig. 4c). We did not find differences between patients and controls in the percentages of viable and apoptotic CD27^–^ B cells (Fig. 4d). In contrast, CD27^+^ B cells showed higher levels of total Caspase-3-activated cells in both CVID patients’ groups compared to controls (Fig. 4e) that nearly statistically correlated with in vitro Bax MFI in CVID CD27^+^ B cells (Spearman’s *R* = 0.683, *P* = 0.0503). Moreover, percentages of viable and intermediate-apoptotic CD27^+^ B cells were significantly lower and higher, respectively, only in AP-CVID patients (Fig. 4f), which indicates a faster spontaneous progression toward irreversible apoptotic stages in these patients. All these findings support our strategy to separately study the AP-CVID group.

### AP-CVID CD27^+^ B cells increase Bcl-2 and Bcl-XL expression at normal levels and reduce cell death in response to anti-CD40 but not anti-BCR stimulation

We evaluated stimulation-induced levels of Bcl-2, Bcl-XL, Bax, and Bim in CD27^–^ and CD27^+^ viable B cells (Fig. 1). Stimulated control B cells differently upregulated Bcl-2 family members, depending on the stimulus and the B cell subpopulation analyzed. Bcl-XL induction was higher in CD27^–^ B cells both after anti-BCR or anti-CD40 stimulation while that of Bcl-2 only after anti-CD40 stimulation. Conversely, Bim induction was higher in CD27^+^ than in CD27^–^ B cells after anti-BCR stimulation (Supplementary Figure 2, panels A and B).

**Fig. 5:**
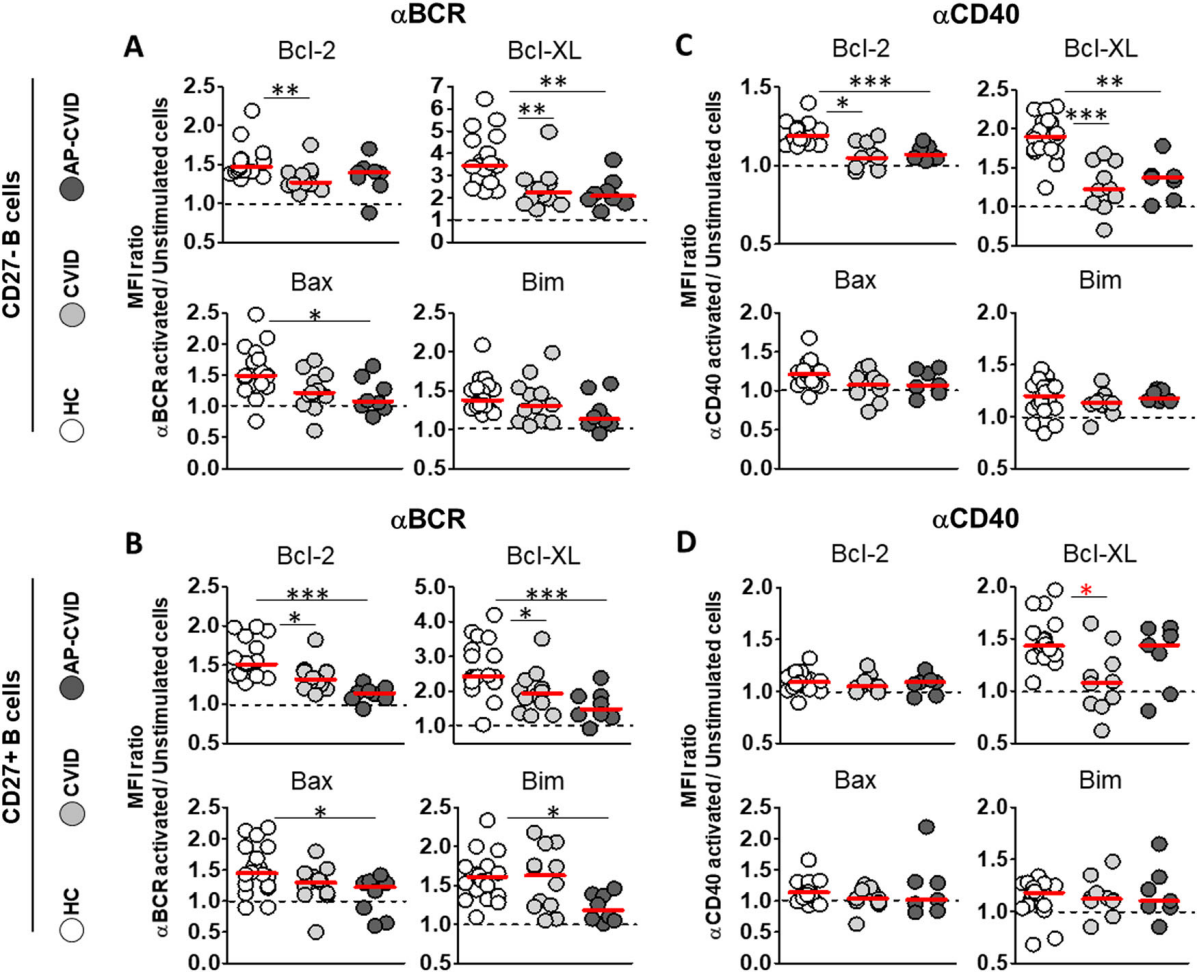
Heterogeneous stimulation-induced expression of Bcl-2 and Bcl-XL between CVID patients after naive and memory B cell activation. MFI ratios of Bcl-2 (upper left), Bcl-XL (upper right), Bax (lower left) and Bim (lower right) in naive CD19^+^CD27^−^ and memory CD19^+^CD27^+^ B cells, unstimulated, and after activation with anti-BCR (**a**, **b**) or anti-CD40 (**c**, **d**) in healthy controls (white circles), CVID (light gray circles), and apoptosis-prone CVID (dark gray circles) patients. Data are given as medians (Kruskal–Wallis test *P* values: *P < 0.05*; **P < 0.01; ***P < 0.001). MFI median fluorescence intensity, HC healthy controls (*n* = 20), CVID common variable immunodeficiency patients (*n* = 12), AP-CVID apoptosis-prone CVID patients (*n* = 8)

When we compared patients and controls after anti-BCR stimulation, AP-CVID CD27^–^ B cells induced lower Bcl-XL and Bax levels and CD27^–^ B cells from the remaining CVID patients induced lower Bcl-2 and Bcl-XL levels (Fig. 5a). In addition, anti-BCR activation of CD27^+^ B cells resulted in lower Bcl-2 and Bcl-XL induced levels in CVID patients and even lower in AP-CVID patients and lower Bax and Bim induced levels only in AP-CVID patients (Fig. 5b). Anti-BCR-induced Bcl-2 and Bcl-XL levels were not enough to counteract its negative effect on in vitro CD27^+^ B cells’ survival, neither in CVID patients nor in controls (Supplementary Figure 3).

Bcl-2 and Bcl-XL levels induced on anti-CD40-stimulated CD27^–^ B cells were lower in both CVID groups than controls (Fig. 5c). However, anti-CD40-induced Bcl-2 levels on CD27^+^ B cells were similar between both CVID groups and controls (Fig. 5d). Interestingly, anti-CD40-stimulated CD27^+^ B cells from AP-CVID patients induced equal Bcl-XL levels than controls, in contrast to the remaining CVID (Fig. 5d). No differences were found on the anti-CD40-induced Bax and Bim expression (Fig. 5c, d). Anti-CD40 activation had a positive effect on the in vitro AP-CVID CD27^+^ B cell survival, just the opposite of what we detected in controls (Supplementary Figure 3).

To study whether observed differences in stimulated B cell survival from controls and CVID patients were due to distinctive apoptosis modulation, we evaluated stimulation-induced Caspase-3 activation and changes in viable and apoptotic CD27^–^ or CD27^+^ B cell percentages (Fig. 2). We found that total Caspase-3 activation increased in control CD27^–^ B cells and decreased in control CD27^+^ B cells both after anti-BCR and anti-CD40 stimulation (Supplementary Figure 4, panels A and B). Accordingly, we observed a decrease of viable and an increase of early-apoptotic CD27^–^ B cells after stimulation. However, we never detected intermediate-apoptotic or late-apoptotic/necrotic CD27^–^ B cells, irrespective of the stimulus used (Fig. 2b and Supplementary Figure 4, panel C). The decrease of total Caspase-3-activated CD27^+^ B cells appeared to be the consequence of the progression from intermediate-apoptotic toward late-apoptotic/necrotic stage after stimulation, rather than to a survival-inducing response (Supplementary Figure 4, panel D). These results suggest that CD27^+^ B cell death commitment is not reverted by activation, due to their advanced apoptosis.

**Fig. 6:**
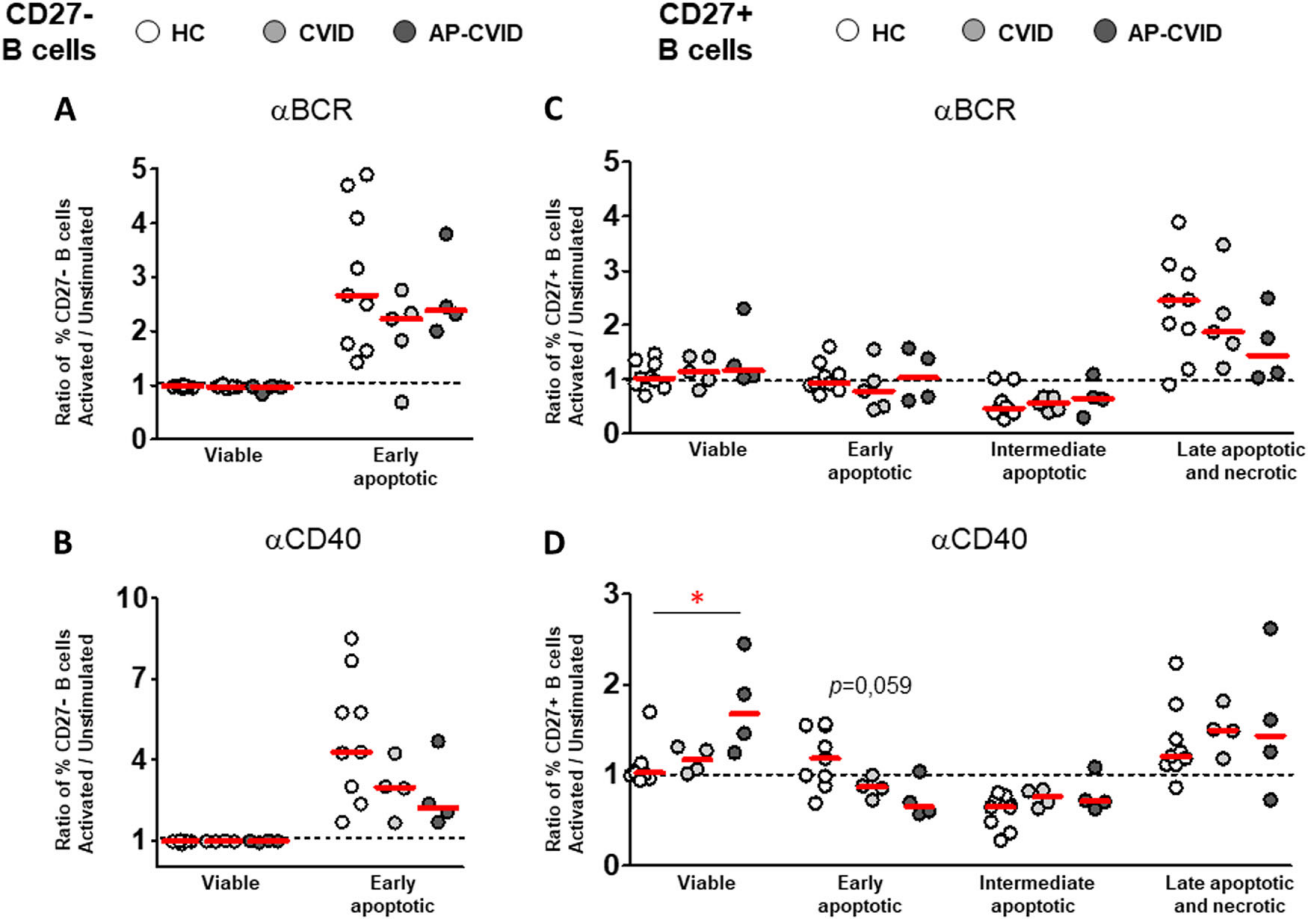
Distinct stimulation-induced levels of viable and apoptotic cells between CVID patients after naive and memory B cell activation. Ratios of percentages of viable and early-apoptotic cells in naive CD19^+^CD27^–^ B cells (left panels) or viable and early-apoptotic, intermediate-apoptotic, and late-apoptotic/necrotic cells in memory CD19^+^CD27^+^ B cells (right panels), unstimulated, and after activation with anti-BCR (**a**, **c**) or anti-CD40 (**b**, **d**) in healthy controls (white circles), CVID (light gray circles), and apoptosis-prone CVID (dark gray circles) patients. Data are given as medians (Kruskal–Wallis test *P* values: *P < 0.05). HC healthy controls (*n* = 9), CVID common variable immunodeficiency patients (*n* = 5), AP-CVID apoptosis-prone CVID patients (*n* = 4)

When patients and controls were compared, we did not find differences in the stimulation-induced levels of viable and apoptotic CD27^–^ B cells, irrespective of the stimulus used, nor in anti-BCR-stimulated CD27^+^ B cells (Fig. 6a–c, respectively). In contrast, levels of viable CD27^+^ B cells significantly increased in AP-CVID patients compared with controls after stimulation with anti-CD40. Moreover, early-apoptotic CD27^+^ B cells decreased after anti-CD40 stimulation mostly in AP patients (Fig. 6d). These results suggest a cell survival recovery induced by anti-CD40 in AP-CVID CD27^+^ B cells.

### IL-21 increases Bcl-XL levels and viability in resting and anti-CD40-stimulated AP-CVID B cells

We evaluated IL-21 effect (alone or combined with anti-BCR or anti-CD40) on control B cell Bcl-2 family protein expression. IL-21 alone had no effect in Bcl-2, Bcl-XL, Bax, or Bim expression neither in CD27^–^ nor in CD27^+^ control B cells (Supplementary Figure 5, panels A and B). When compared to anti-BCR alone, anti-BCR+IL-21 combination diminished CD27^–^ B cell Bcl-2 and Bcl-XL levels and CD27^+^ B cell Bcl-2 levels (Supplementary Figure 5, panels A and B), which demonstrates that IL-21 cell death triggered in BCR-ligated B cells is influenced by downmodulation of survival-inducing signals. In contrast, when we compared anti-CD40+IL-21 combination to anti-CD40 alone, we observed different effects depending on the B cell subpopulation and the anti-apoptotic protein evaluated. Thus anti-CD40+IL-21 diminished CD27^–^ B cell Bcl-2 levels but increased CD27^+^ B cell Bcl-XL levels (Supplementary Figure 5, panels A and B). Regarding pro-apoptotic protein modulation, both anti-BCR+IL-21 and anti-CD40+IL-21 combinations showed an inducing effect, greater in CD27^–^ B cells (Supplementary Figure 5, panels A and B).

**Fig. 7:**
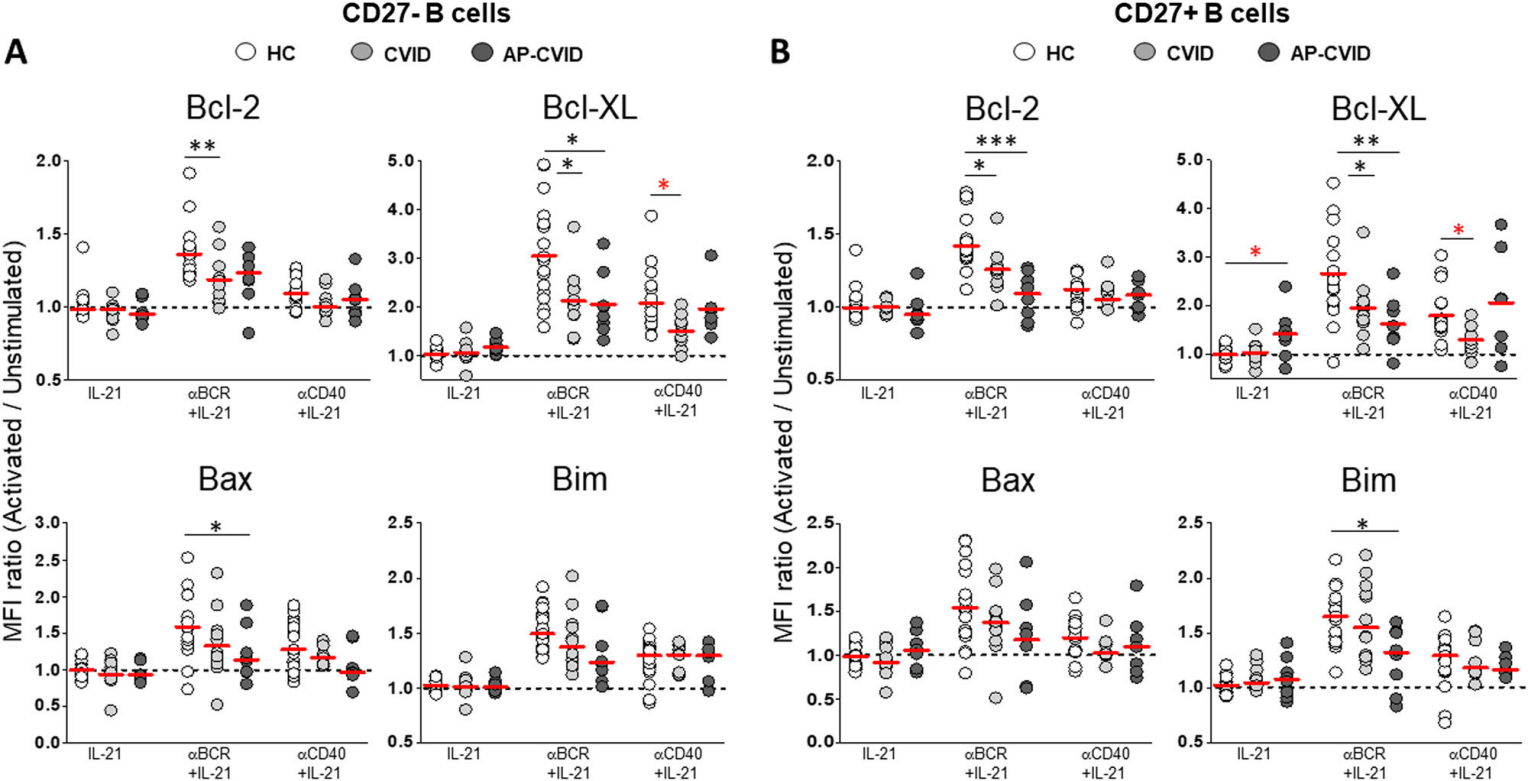
Higher Bcl-XL expression induced by IL-21 co-stimulation in B cells from apoptosis-prone CVID patients. MFI ratios of Bcl-2, Bcl-XL (upper panels), Bax, and Bim (lower panels) in naive CD19^+^CD27^–^ B cells (**a**) and memory CD19^+^CD27^+^ B cells (**b**), unstimulated and after activation with IL-21 alone (left), anti-BCR+IL-21 (middle), or anti-CD40+IL-21 (right), in healthy controls (white circles), CVID (light gray circles), and apoptosis-prone CVID (dark gray circles) patients. Data are given as medians (Mann–Whitney and Kruskal–Wallis test *P* values: *P < 0.05; **P < 0.01; ***P < 0.001). MFI median fluorescence intensity, HC healthy controls (*n* = 20), CVID common variable immunodeficiency patients (*n* = 12), AP-CVID apoptosis-prone CVID patients (*n* = 8)

When we compared patients and controls, we found higher Bcl-XL levels induced by IL-21 alone only in AP-CVID CD27^+^ B cells (Fig. 7b). After anti-BCR+IL-21 stimulation, both CD27^–^ and CD27^+^ B cells from AP-CVID patients and remaining CVID induced lower Bcl-2 and Bcl-XL levels than controls (Fig. 7a, b). In addition, anti-BCR+IL-21 induced lower levels of Bax and Bim, respectively, on AP-CVID CD27^–^ and CD27^+^ B cells (Fig. 7a, b). Anti-BCR+IL-21 stimulation had a negative effect on B cell survival in vitro, both in control CD27^–^ and CD27^+^ B cells and in CVID CD27^+^ B cells (Supplementary Figure 6).

Interestingly, anti-CD40+IL-21 stimulation of CD27^–^ B cells induced normal Bcl-2 levels in both CVID groups and normal levels of Bcl-XL in AP patients (Fig. 7a), contrarily to what happened in anti-CD40-stimulated CD27^–^ B cells (Fig. 5c). Conversely, Bcl-2 and Bcl-XL were similarly induced by anti-CD40+IL-21 or anti-CD40 alone in CD27^+^ B cells (Figs. 7b and 5d). We did not find differences between controls and patients in anti-CD40+IL-21-induced Bax or Bim neither in CD27^–^ nor in CD27^+^ B cells (Fig. 7a, b). These results demonstrate a positive effect on in vitro induction of anti-apoptotic signals in CVID B cells when both cell-to-cell contact and T-derived soluble factors are properly provided. This translated into in vitro survival preservation of anti-CD40+IL-21-stimulated AP-CVID CD27^+^ B cells (Supplementary Figure 6).

When we studied different apoptosis stages, we found that IL-21 alone, anti-BCR+IL-21, and anti-CD40+IL-21 increased total Caspase-3-activated control CD27^–^ B cells, what was mirrored by an increase of early-apoptotic cells. Again, we did not detect intermediate or late-apoptotic/necrotic CD27^–^ B cells regardless of IL-21 (Supplementary Figure 7, panels A and B). In contrast, the decrease of Caspase-3-activated control CD27^+^ B cells induced by IL-21 alone, anti-BCR+IL-21, and anti-CD40+IL-21 was explained once more by a late-apoptotic/necrotic cell increase (Supplementary Figure 7, panels A and C).

**Fig. 8:**
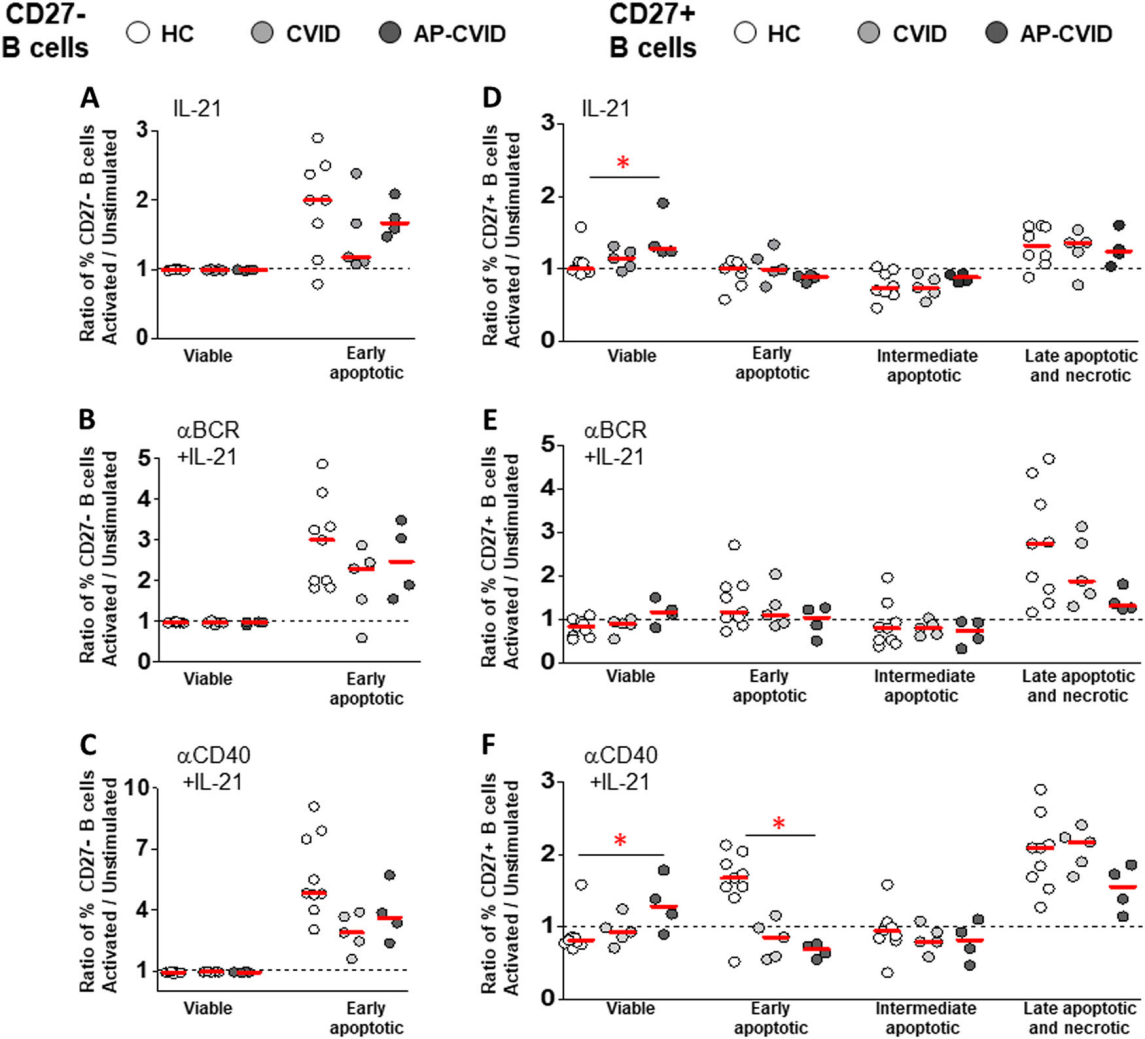
Pro-survival effect of IL-21 co-stimulation on viable and apoptotic memory B cell subpopulations from apoptosis-prone CVID patients. Ratios of percentages of viable and early-apoptotic cells in naive CD19^+^CD27^–^ B cells (left panels) or viable and early-apoptotic, intermediate-apoptotic, and late-apoptotic/necrotic cells in memory CD19^+^CD27^+^ B cells (right panels), unstimulated and after activation with IL-21 alone (**a**, **d**), anti-BCR+IL-21 (**b**, **e**), or anti-CD40+IL-21 (**c**, **f**), in healthy controls (white circles), CVID (light gray circles), and apoptosis-prone CVID (dark gray circles) patients. Data are given as medians (Kruskal–Wallis test *P* values: *P < 0.05). HC healthy controls (*n* = 9), CVID common variable immunodeficiency patients (*n* = 5); AP-CVID apoptosis-prone CVID patients (*n* = 4)

When patients and controls were compared, we found a distinctive activation-induced apoptosis modulation only in AP-CVID CD27^+^ B cells (Fig. 8). Viable CD27^+^ B cells significantly increased after stimulation with both IL-21 alone and anti-CD40+IL-21, and early-apoptotic CD27^+^ B cells decreased after anti-CD40+IL-21 stimulation in these patients (Fig. 8d, f).

## Discussion

Survival, growth, and differentiation signals are required to maintain B cell homeostasis and induce differentiation into memory B cells and ASC. Autophagy and mitochondrial functions interplay controls B cell development and memory B cell maintenance^26^. Mitochondria control cell death by releasing apoptosis-related signals. The impairment of B cell apoptosis regulation has been already proposed as a CVID and selective IgA deficiency pathogenic mechanism^15,27^. Despite current knowledge recently reviewed by Yazdani et al.^28^, molecular mechanisms underlying defective protection from cell death in CVID have not been elucidated. Thus we evaluated mitochondrial apoptosis regulation induced by Bcl-2 family proteins in CVID. We report increased basal Bax and Bim pro-apoptotic levels in CVID patients that correlated with low survival of their CD27^+^ B cells. Regarding activation-induced Bcl-2 and Bcl-XL anti-apoptotic levels, we detected a broad B cell defect in CVID, irrespective of the stimulus.

High ex vivo CD27^+^ B cell Bim levels were particularly detected in a CVID subgroup with disturbed B cell memory and high prevalence of cytopenias. After unstimulated culture, viable CD27^+^ B cell percentages were lower and Caspase-3-activated CD27^+^ B cells higher in this subgroup classified as AP-CVID. In fact, ex vivo CVID CD27^+^ B cell Bax and Bim levels correlated with their lower survival in vitro. In addition, in vitro high levels of Bax (intrinsic apoptosis pathway executor) in unstimulated CVID CD27^+^ B cells correlated with higher spontaneous Caspase-3 activation. These results confirm that the survival/apoptosis equilibrium in AP-CVID CD27^+^ B cells is constitutively unbalanced toward cell death.

Although we detected higher ex vivo CVID CD27^–^ B cell Bim levels, this was not accompanied by a spontaneous Caspase-3 activation or an in vitro survival decrease. We have previously shown that unstimulated CD27^–^ B cells die from apoptosis, although to a lesser extent than CD27^+^ B cells^15^. We hypothesize that the effect of pro-apoptotic imbalance in CD27^–^ B cells is delayed with regard to CD27^+^ B cells. In keeping with this, we also demonstrated that only CD27^+^ B cells from CVID patients exhibited baseline higher pro-apoptotic membrane TNF-related apoptosis-inducing ligand (TRAIL) expression^15^. Higher TRAIL expression has been related to peripheral memory B cell apoptosis in successfully treated aviremic HIV patients^29^ and loss of these cells during HIV infection was associated with increased IL-21R expression, low Bcl-2, and high Bim levels^30^. However, as distinct relative levels of anti-apoptotic Bcl-2, MCL-1, and A1 proteins influence survival depending on immune cell subset^31^, we cannot rule out other factors as additional protective signals to CD27^–^ B cells.

In this study, we also evaluated stimulation-induced levels of Bcl-2 family proteins in B cells after anti-BCR or anti-CD40 activation with or without IL-21. Keller et al. previously reported impaired BCR-mediated Bcl-XL induction in naive B cells from CVID patients with expanded CD21^low^ cells^32^. Our present results demonstrate a broad CVID B cell defect regarding Bcl-2 and Bcl-XL upregulation as an apoptosis prevention mechanism. This was true in all CVID patients for both anti-BCR-activated CD27^–^ and CD27^+^ B cells and for anti-CD40-activated CD27^–^ B cells, irrespective of IL-21 effect. These findings suggest that increased apoptosis during B cell immune responses could result in disrupted B cell differentiation to memory and ASC in CVID. In fact, impaired DNA repair mechanisms during somatic hypermutation and Ig genes rearrangements in CVID result in DNA damage^33^, an intrinsic apoptosis trigger. Interestingly, anti-CD40 and IL-21 induced normal and even higher Bcl-XL levels respectively in AP-CVID CD27^+^ B cells, mirrored by a cell viability increase. These results agree with previous findings by Borte et al. reporting that IL-21 reduced apoptosis in anti-CD40-stimulated CVID naive and memory B cells and restored Ig production in vitro^34^.

We hypothesize that CD4 T cell-mediated B cell apoptosis rescue is altered in AP-CVID patients, given that their CD27^+^ B cells respond properly to T-surrogate stimuli in vitro. Apparently, the presumptive lack of T-dependent survival signals to B cells was not due to a decrease of circulating CD4 T cell frequency in our AP-CVID cohort (Supplementary Tables 1 and 2). However, increased apoptosis of CVID CD4 T cells has been previously reported^35,36^. In addition, follicular T helper (Tfh) cells, specialized in directing B cell differentiation, have been previously studied in several CVID cohorts including ours^37,38^, demonstrating an increase of “non-B cell helper” circulating Tfh1 subset in CVID patients. Moreover, we also reported high levels of programmed death (PD)-1 (T cell helper function inhibitor), in circulating Tfh from CVID patients with compromised memory B cell compartment. In order to relate previous with present results, we retrospectively analyzed Tfh cell PD-1 expression in AP-CVID patients and detected a PD-1^+^ cell percentage increase (median and 25th–75th percentiles: 7.4% and 5.8–9.2% in controls versus 17.3% and 10.4–23.4% in AP-CVID versus 8.4% and 6.0–15.6% in remaining CVID patients; Kruskal–Wallis test *P* < 0.01). Thus inhibitory functional changes in circulating Tfh cells may contribute to defective T-related B cell apoptosis protection in AP-CVID patients.

Alternatively, restriction of T–B encounters in vivo might underlie the lack of T cell collaboration. In fact, we found higher levels of AP-CVID B cells lacking surface expression of chemokine receptor CXCR5, that mediates migration of B cells to lymph nodes follicular zone (Cunill et al. unpublished results). Inefficient germinal centers (GC) responses, GC with irregular shapes and hyperplasia, have been described in a CVID subgroup with cytopenias and severe isotype-switched memory B cell reduction (features shared by our AP-CVID)^39^. Thus AP-CVID B cell apoptosis commitment could be also influenced by limited T–B encounters, due to abnormal GC generation and/or decreased ability of B cells to recirculate between blood and lymph nodes, leading to an inactivated status. This is supported by Liu et al. study using a murine model, demonstrating that Bim (constitutively increased in AP-CVID) plays a critical role in B cell homeostasis limiting non-activated mature follicular B cell survival^40^.

Despite constitutive defects detected in AP-CVID patients regarding pro-apoptotic molecule expression, their CD27^+^ B cells recover pro-survival balance, via Bcl-2 and Bcl-XL upregulation, if properly activated in vitro. Final AP-CVID CD27^+^ B cell death reversibility is the result of early-apoptotic cell rescue. In keeping with this, Geske et al. reported that cells within early apoptosis stage can be rescued from the apoptotic program^41^. In the remaining CVID, a more profound B cell defect may cause apoptosis dysregulation, since their B cells do not respond normally neither to anti-BCR nor to T-dependent stimuli. Non-evaluated Bcl-2 family members or defective long-term memory B cell maintenance control by autophagy^42^ could contribute to the heterogeneous B cell apoptosis regulation among CVID.

The heterogeneity of CVID B cell responses has been revealed by in vitro studies of Ig production. We previously reported impaired but still detectable Ig secretion in purified B cell cultures stimulated with anti-CD40 or TLR-9 ligand in 15 CVID patients irrespective of IL-21 influence^43^, and Borte et al. demonstrated that IL-21 restores Ig production by PBMCs in 14 CVID patients^34^. These original studies did not identify patients’ subgroups with different B cell Ig secretion capabilities. Addressing this concern, Rösel et al. proposed a new CVID classification based on distinct memory B cell capabilities to develop into ASC in 14 CVID^19^. An almost normal ASC functionality characterized a “responder” subgroup in contrast to “non-responders” and “low responders”. Moreover, Desjardins et al. used an in vitro assay stimulating PBMCs with anti-CD40 combined with IL-21 and/or IL-4 in a 42 CVID patients’ cohort^20^. By measuring CD27^+^ memory B cell development and IgG secretion, IL-21 pathway discriminated subgroups of “responder” and “non-responder”. In both studies, CVID patients with lower switched memory B cells were the worst responders. Finally, Gardulf et al. evaluated in vivo response to T-dependent influenza vaccine Pandemrix in 48 CVID patients^44^ and distinguished a “responder” subgroup with post-GC B cell pattern in blood in contrast to “non-responders” with progressive antibody deficiency. Interestingly, patients with cytopenias were exclusively “non-responders”, analogously to our AP patients with presumed specific T cell fail or restricted T–B contacts in vivo. Despite the main CVID immunological defect is a failure of Ig production by B cells, a plethora of immune cell abnormalities have been described and associated with clinical variability^3,4^. Here we underscore distinct molecular mechanisms underlying intrinsic CVID B cell differentiation defects, although we cannot completely exclude other abnormal populations influencing B cell apoptosis regulation.

We hypothesize that CVID B cell functional heterogeneity could be due in part to differential sensibility to apoptosis. B cell counts and case history of cytopenias might be useful to consider specific vaccination strategies with T-derived adjuvants or predict positive responses to therapeutic approaches targeting T-dependent signaling pathways. The study of specific apoptosis markers could provide additional evidence to better characterize CVID patients based on B cell responses.

## Electronic supplementary material

Supplementary Table 1

Supplementary Table 2

Supplementary Figure 1. Heterogeneous basal levels of Bcl-2 family proteins between control naïve and memory B cells

Supplementary Figure 2. Different stimulation-induced levels of Bcl-2 family proteins between control naïve and memory B cells

Supplementary Figure 3. B cells in vitro survival is differently influenced by stimulation between healthy controls and CVID patients

Supplementary Figure 4. Distinct stimulation-induced levels of viable and apoptotic cells between control naïve and memory B cells

Supplementary Figure 5. Bcl-2 family proteins expression is distinctively modulated by IL-21 co-stimulation in control naïve and memory B cells

Supplementary Figure 6. B cells in vitro survival is differently influenced by IL-21 co-stimulation between healthy controls and CVID patients

Supplementary Figure 7. Distinct IL-21-induced levels of viable and apoptotic cells between control naïve and memory B cells

Supplementary figure legends
