# Supplementary material for: IL-21 and anti-CD40 restore Bcl-2 family protein imbalance in vitro in low-survival CD27+ B cells from CVID patients
Source: Cell Death Dis. 2018 Nov 21;9(12):1156. doi: 10.1038/s41419-018-1191-8 (PMC6249202; doi:10.1038/s41419-018-1191-8)
Supplement: Supplementary file 1 — Supplementary Table 1 [file 41419_2018_1191_MOESM1_ESM.doc]

| **Patient** | **Age**  years | **Age at diagnosis**  years | **Gender** | **IgG**  mg/dl | **IgA**  mg/dl | **IgM**  mg/dl | **CD19**  (%) | **CD19+** (%) | | | | **EURO**  **class** | **CD19+** | | **CD4**  (cells/µL) | **Autoimmune**  **manifestations** | **Enteropathy**  **symptoms** | **Lymphoproliferative complications** | **Malignancy** |
| --- | --- | --- | --- | --- | --- | --- | --- | --- | --- | --- | --- | --- | --- | --- | --- | --- | --- | --- | --- |
|  |  |  |  |  |  |  |  | **CD21**  **low** | **IgD+**  **CD27-** | **IgD+ CD27+** | **IgD-CD27+** |  | **CD27-**  (cells/µL) | **CD27+**  (cells/µL) |  |  |  |  |  |
| 1 | 67 | 55 | M | 316 | 9 | 12 | 6 | 19 | 91 | 7 | <1 | smB- | NA | NA | 1093 | Haemolytic anaemia  Thrombopenia | - | Nodular regenerative hyperplasia | B cell  Lymphoma |
| 2 | 65 | 49 | F | 112 | <6 | <5 | 6 | 16 | 87 | 7 | <1 | smB- | 69 | 13 | 734 | - | + | - | - |
| 3 | 65 | 52 | M | 77 | <6 | <4 | 2 | 26 | 86 | 7 | <2 | smB- | 28 | 4 | 424 | - | - | - | - |
| 4 | 72 | 66 | F | 37 | <6 | <5 | 4 | 33 | 82 | 5 | <1 | smB- | 21 | 3 | 397 | Thrombopenia | - | Splenomegaly | - |
| 5 | 33 | 25 | F | 330 | 107 | 96 | 12 | 10 | 92 | 5 | <1 | smB- | 204 | 10 | 442 | Neutropenia  Thrombopenia | - | Nodular regenerative hyperplasia | - |
| 6 | 35 | 15 | M | 229 | <6 | 12 | 4 | 66 | 91 | 1 | 1 | smB- | 7 | 1 | 308 | Haemolytic anaemia Thrombopenia | + | - | - |
| 7 | 47 | 40 | F | 772* | <7* | <5* | 7 | 41 | 97 | 1 | <1 | smB- | 46 | 1 | 305 | Thrombopenia | - | GLILD | - |
| 8 | 88 | 72 | F | 253 | 26 | <16 | 2 | 20 | 39 | 14 | 36 | smB+ | 13 | 11 | 215 | Vitiligo | - | - | - |
| 9 | 44 | 36 | M | 85 | <24 | <18 | 6 | 13 | 89 | 7 | <1 | smB- | 95 | 41 | 702 | Lichen  Planus | - | Nodular lymphoid hyperplasia | - |
| 10 | 34 | 30 | F | 164 | <7 | 8 | 24 | 5 | 89 | 6 | <2 | smB- | 128 | 48 | 490 | - | + | - | - |
| 11 | 79 | 67 | F | 323 | 98 | <6 | 24 | 55 | 73 | 26 | <1 | smB- | 113 | 23 | 575 | - | - | - | - |
| 12 | 28 | 22 | M | 316 | <25 | 21 | 14 | 35 | 91 | 4 | <2 | smB- | 247 | 106 | 438 | - | - | - | - |
| 13 | 38 | 28 | M | 75 | <25 | <17 | 12 | 34 | 82 | 10 | <2 | smB- | NA | NA | 677 | - | + | - | - |
| 14 | 33 | 16 | M | 461 | <28 | <17 | 17 | 17 | 87 | 7 | <1 | smB- | NA | NA | 222 | - | + | Nodular lymphoid hyperplasia | - |
| 15 | 49 | 36 | F | 288 | 32 | 14 | 20 | 6 | 55 | 13 | 17 | smB+ | 214 | 126 | 603 | - | + | - | - |
| 16 | 66 | 50 | F | 327 | 73 | 30 | 20 | 5 | 70 | 15 | 9 | smB+ | 411 | 109 | 1045 | CLE | + | - | - |
| 17 | 72 | 55 | F | 452 | 46 | 58 | 7 | 5 | 47 | 13 | 19 | smB+ | 30 | 55 | 788 | - | - | - | - |
| 18 | 45 | 40 | F | 387 | <7 | 17 | 6 | 8 | 70 | 11 | 10 | smB+ | 200 | 70 | 744 | - | + | - | - |
| 19 | 35 | 20 | M | 82 | <6 | 5 | 10 | 11 | 94 | 3 | <2 | smB- | 393 | 30 | 711 | - | - | - | - |
| 20 | 35 | 21 | F | 351 | 20 | <6 | 15 | 24 | 81 | 11 | 2 | smB- | 207 | 52 | 616 | - | - | Nodular lymphoid hyperplasia | - |

**Supplementary Table 1.** Age, gender, immunoglobulin levels, B cells subpopulations, absolute counts of CD4 T cells, autoimmune manifestations, enteropathy presence, lymphoproliferative complications and malignancies of CVID patients.

Current age (years) and gender (M: male, F: female). Seric immunoglobulin levels (IgG, IgA and IgM) before starting replacement therapy; percentage of peripheral blood B cells (CD19^+^), percentages of CD21low, naïve (IgD^+^CD27^−^), unswitched memory (IgD^+^CD27^+^) and switched memory (IgD^−^CD27^+^) B-cells subpopulations (referred to total CD19^+^ B-cells) and EUROclass classification (smB-: ≤2% of IgD^–^CD27^+^ B cells; smB+: >2% of IgD^–^CD27^+^ B cells) at diagnosis. Absolute counts of naïve B cells (CD19^+^CD27^−^) total memory B cells (CD19^+^CD27^+^) and total T cells (CD4^+^) at the time of the study. Autoimmune manifestations, enteropathy presence, lymphoproliferative complications and malignancies of CVID patients. NA: not available; CLE: Cutaneous Lupus Erythematosus; GLILD: Granulomatous and Lymphocytic Interstitial Lung Disease. *Values after replacement therapy.
