# Supplementary material for: IL-21 and anti-CD40 restore Bcl-2 family protein imbalance in vitro in low-survival CD27+ B cells from CVID patients
Source: Cell Death Dis. 2018 Nov 21;9(12):1156. doi: 10.1038/s41419-018-1191-8 (PMC6249202; doi:10.1038/s41419-018-1191-8)
Supplement: Supplementary file 2 — Supplementary Table 2 [file 41419_2018_1191_MOESM2_ESM.docx]

**Supplementary Table 2. Clinical characteristics of apoptosis-prone CVID patients.**

|  | **Apoptosis-prone CVID**  (n=8) | **CVID**  (n=12) | ***p* value** |
| --- | --- | --- | --- |
|  | **n** (%) | **n** (%) |  |
| **< 5% of CD19^+^ B cells** | **4** (50.0) | **0** (0) | 0.014* |
| **< 2% of switched memory B cells** | **7** (87.5) | **7** (58.3) | 0.324 |
| **CD4 T lymphopenia (<400 cells/μL)** | **3** (37.5) | **1** (8.3) | 0.117 |
| **Enteropathy symptoms** | **2** (25.0) | **6** (50.0) | 0.373 |
| **Lymphoproliferative disorders** | **4** (50.0) | **3** (25.0) | 0.356 |
| **Malignancy** | **1** (12.5) | **0** (0) | 0.400 |
| **Autoimmune manifestations** | **6** (75.0) | **2** (16.7) | 0.019* |
| **Only infections** | **1** (12.5) | **4** (33.3) | 0.603 |
| **≥ 2 non-infectious complications** | **5** (62.5) | **3** (25.0) | 0.160 |

Percentages of circulating CD19^+^ B cells and switched memory B cells, CD4 T lymphopenia and clinical manifestations of apoptosis-prone CVID patients *versus* remaining CVID patients. Statistical analyses were performed by Fisher exact test. **p* value <0.05.
