# Supplementary figures and images for: IL-21 and anti-CD40 restore Bcl-2 family protein imbalance in vitro in low-survival CD27+ B cells from CVID patients
Source: Cell Death Dis. 2018 Nov 21;9(12):1156. doi: 10.1038/s41419-018-1191-8 (PMC6249202; doi:10.1038/s41419-018-1191-8)

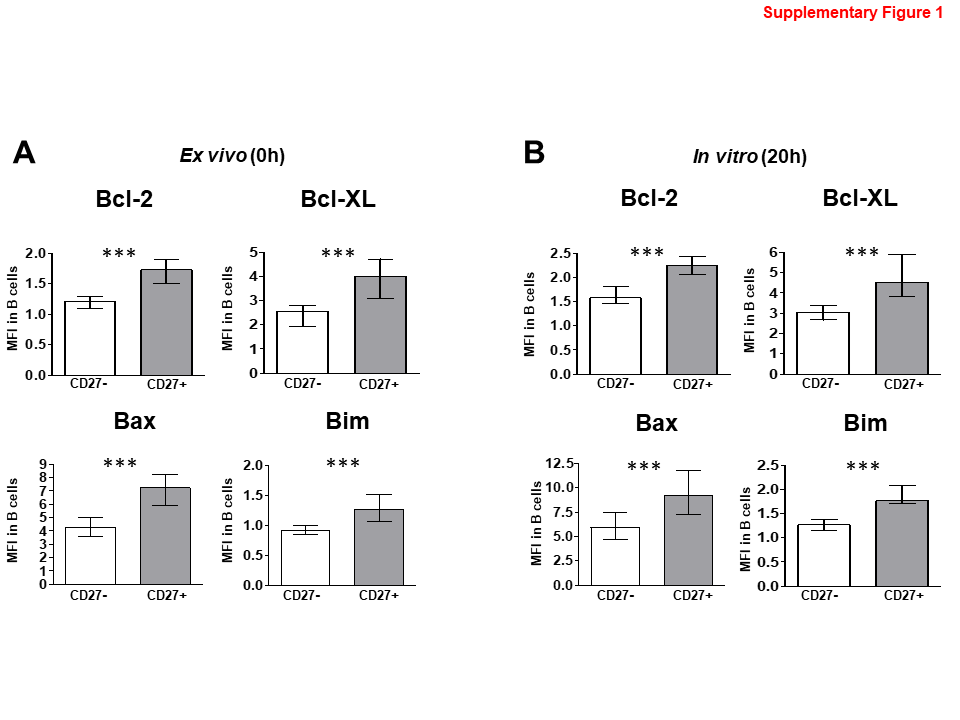

Supplement: Supplementary file 3 — Supplementary Figure 1. Heterogeneous basal levels of Bcl-2 family proteins between control naïve and memory B cells [file 41419_2018_1191_MOESM3_ESM.tif]

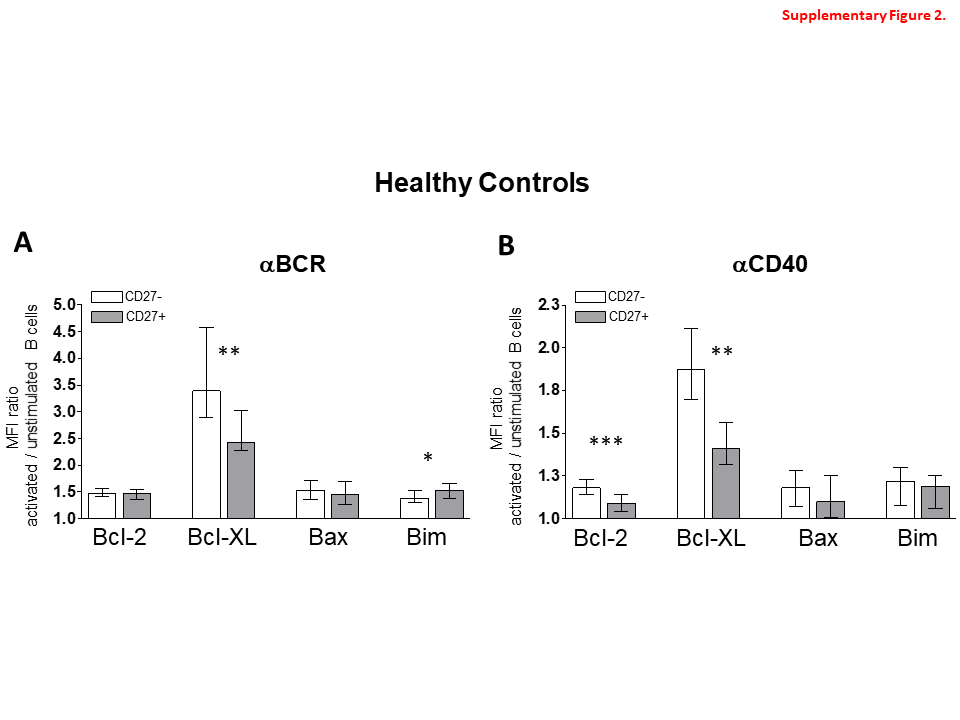

Supplement: Supplementary file 4 — Supplementary Figure 2. Different stimulation-induced levels of Bcl-2 family proteins between control naïve and memory B cells [file 41419_2018_1191_MOESM4_ESM.tif]

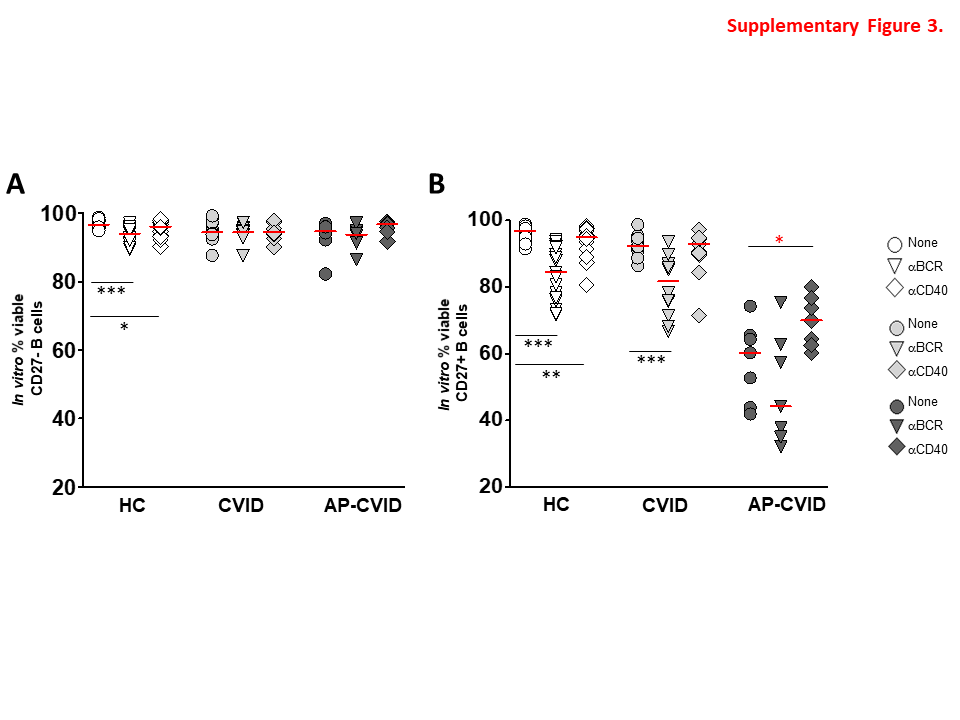

Supplement: Supplementary file 5 — Supplementary Figure 3. B cells in vitro survival is differently influenced by stimulation between healthy controls and CVID patients [file 41419_2018_1191_MOESM5_ESM.tif]

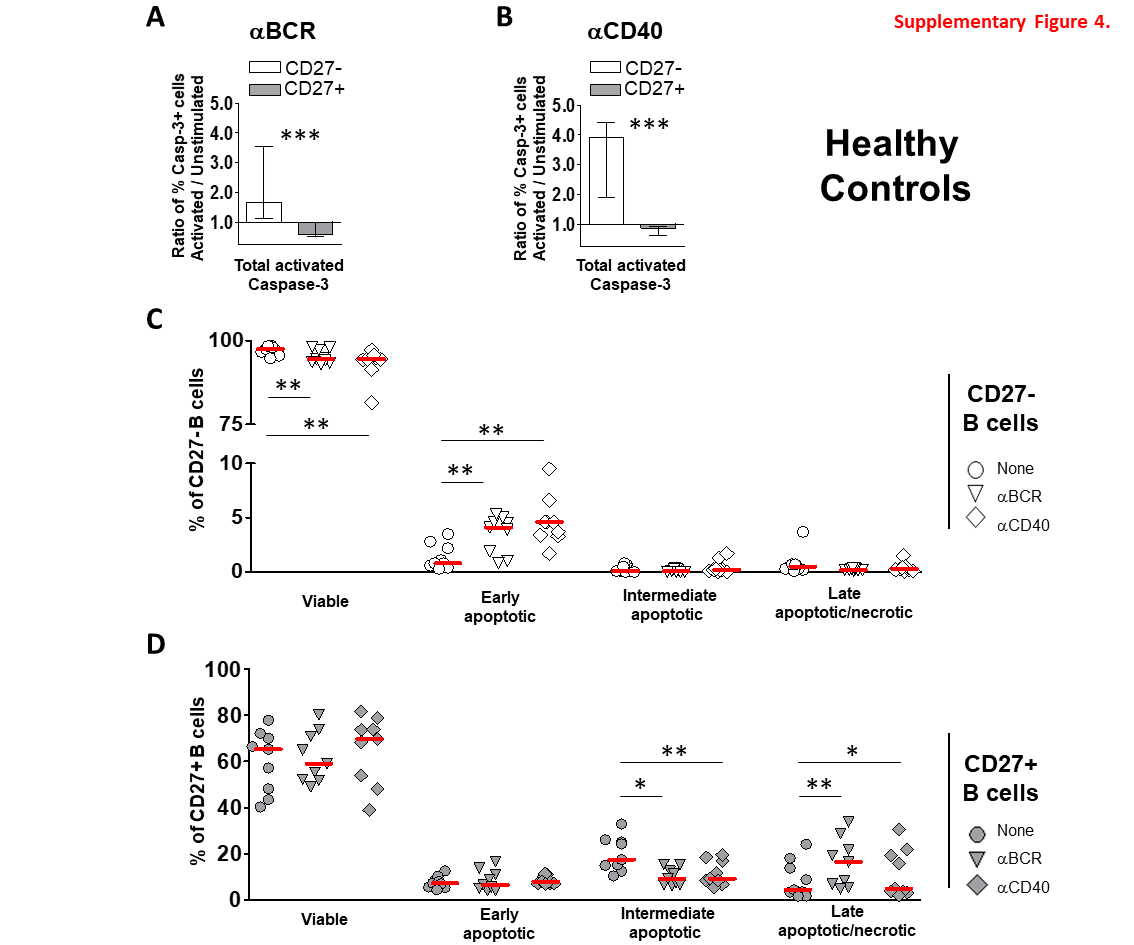

Supplement: Supplementary file 6 — Supplementary Figure 4. Distinct stimulation-induced levels of viable and apoptotic cells between control naïve and memory B cells [file 41419_2018_1191_MOESM6_ESM.tif]

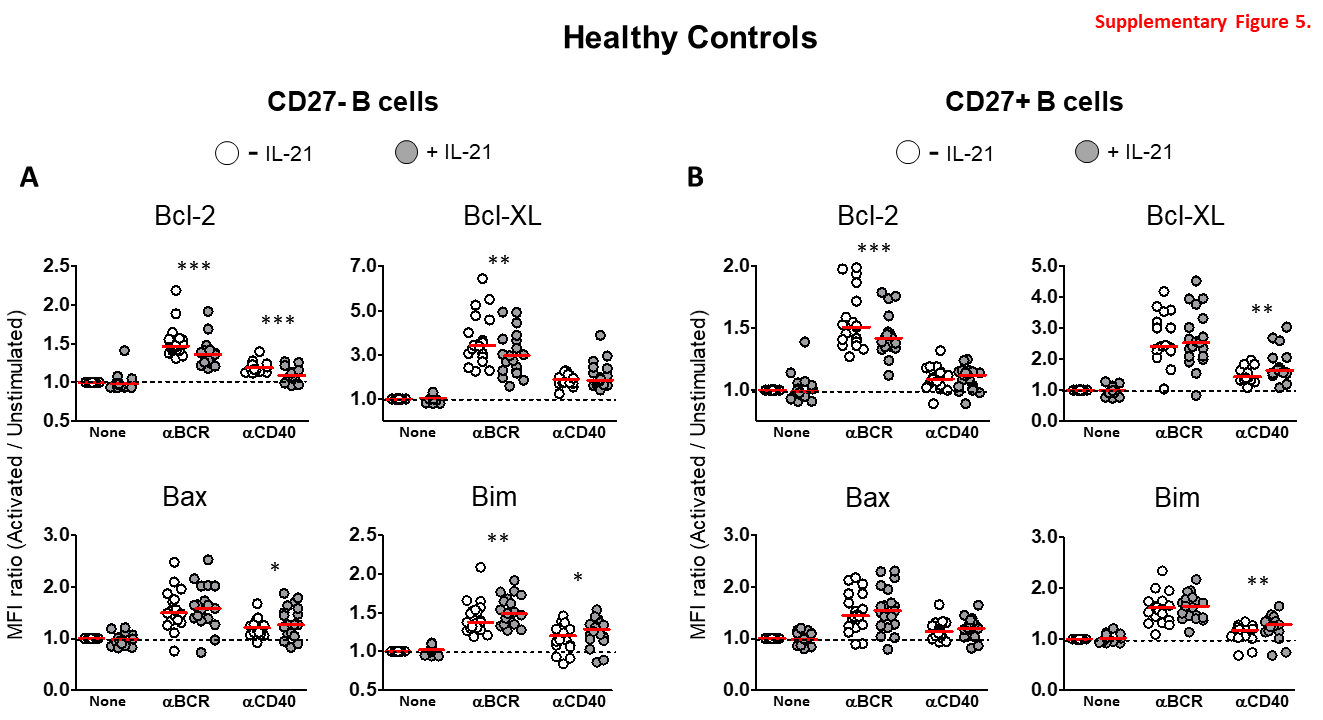

Supplement: Supplementary file 7 — Supplementary Figure 5. Bcl-2 family proteins expression is distinctively modulated by IL-21 co-stimulation in control naïve and memory B cells [file 41419_2018_1191_MOESM7_ESM.tif]

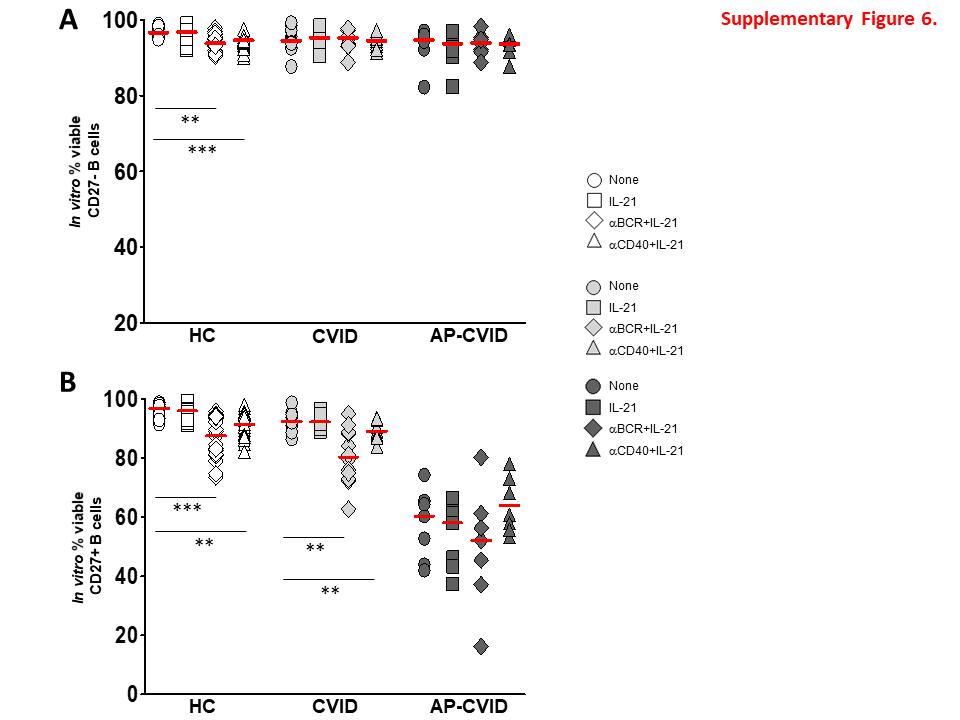

Supplement: Supplementary file 8 — Supplementary Figure 6. B cells in vitro survival is differently influenced by IL-21 co-stimulation between healthy controls and CVID patients [file 41419_2018_1191_MOESM8_ESM.tif]

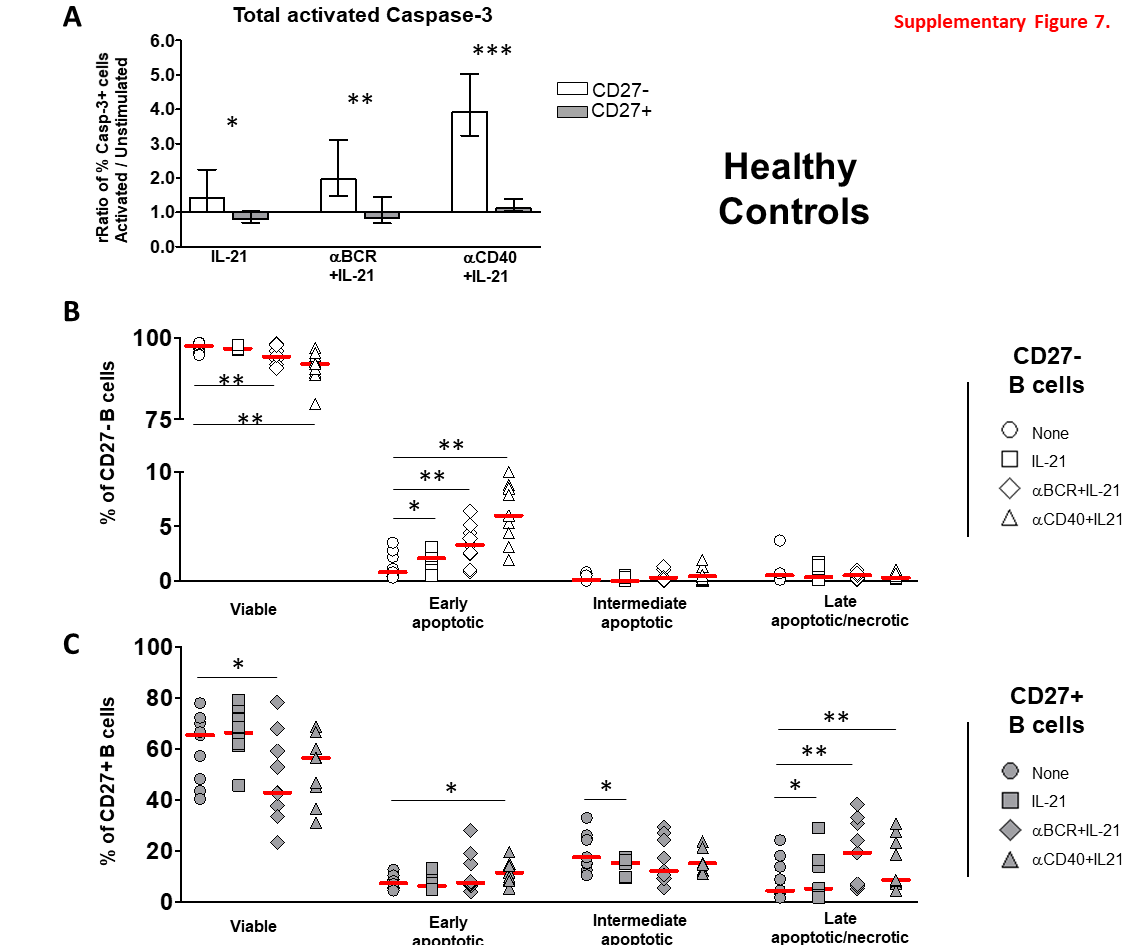

Supplement: Supplementary file 9 — Supplementary Figure 7. Distinct IL-21-induced levels of viable and apoptotic cells between control naïve and memory B cells [file 41419_2018_1191_MOESM9_ESM.tif]
