# Supplementary material for: IL-21 and anti-CD40 restore Bcl-2 family protein imbalance in vitro in low-survival CD27+ B cells from CVID patients
Source: Cell Death Dis. 2018 Nov 21;9(12):1156. doi: 10.1038/s41419-018-1191-8 (PMC6249202; doi:10.1038/s41419-018-1191-8)
Supplement: Supplementary file 10 — Supplementary figure legends [file 41419_2018_1191_MOESM10_ESM.docx]

**SUPPLEMENTARY FIGURE LEGENDS**

**Supplementary Figure 1. Heterogeneous basal levels of Bcl-2 family proteins between control naïve and memory B-cells.**

MFI levels of Bcl-2 (upper left), Bcl-XL (upper right), Bax (lower left) and Bim (lower right) in naïve CD19+CD27− (white bars) and memory CD19+CD27+ (grey bars) B-cells from controls detected in ex vivo freshly isolated cells (Panels A) and in vitro (Panels B) cultured cells without stimulation (Panels C and D). Data are given as medians and 25-75th percentiles from 20 independent experiments (Mann–Whitney test P-values: P<0.001***). MFI: median fluorescence intensity.

**Supplementary Figure 2. Different stimulation-induced levels of Bcl-2 family proteins between control naïve and memory B-cells.**

MFI ratios of Bcl-2, Bcl-XL, Bax and Bim in naïve CD19^+^CD27^−^ (white bars) and memory CD19^+^CD27^+^ (grey bars) control B-cells, unstimulated and after activation with anti-BCR (Panel A) or anti-CD40 (Panel B). Data are given as medians and 25-75th percentiles from 20 independent experiments (Mann–Whitney test P-values: P<0.05*; P<0.01**; P<0.001***). MFI: median fluorescence intensity.

**Supplementary Figure 3. B-cells *in vitro* survival is differently influenced by stimulation between healthy controls and CVID patients.**

Percentages of viable (Viability Dye-eFluor520 negative) naïve CD19^+^CD27^−^ (Panel A) and memory CD19^+^CD27^+^ (Panel B) B-cells unstimulated (circles) and after activation with anti-BCR (triangles) or anti-CD40 (rhombus) in controls (white), CVID (light grey) and apoptosis-prone CVID (dark grey) patients. Data are given as medians (Wilcoxon test P-values: P<0.05*; P<0.01**; P<0.001***). HC: healthy controls (n=20); CVID: Common Variable Immunodeficiency patients (n=12); AP-CVID: apoptosis-prone CVID patients (n=7).

**Supplementary Figure 4. Distinct stimulation-induced levels of viable and apoptotic cells between control naïve and memory B-cells.**

Ratios of percentage of total Caspase-3-activated naïve CD19^+^CD27^−^ (white bars) and memory CD19^+^CD27^+^ (grey bars) control B-cells, unstimulated and after activation with anti-BCR (Panel A) or anti-CD40 (Panel B). Data are given as medians and 25-75th percentiles from 9 independent experiments (Mann–Whitney test P-values: P<0.001***). Percentages of viable, early-apoptotic, intermediate-apoptotic and late-apoptotic/necrotic cells in naïve CD19^+^CD27^−^ (Panel C) and memory CD19^+^CD27^+^ (Panel D) control B-cells unstimulated (circles) and after activation with anti-BCR (triangles) or anti-CD40 (rhombus). Data are given as medians from 9 independent experiments (Wilcoxon test P-values: P<0.05*; P<0.01**).

**Supplementary Figure 5. Bcl-2 family proteins expression is distinctively modulated by IL-21 co-stimulation in control naïve and memory B-cells.**

MFI ratios of Bcl-2, Bcl-XL, Bax and Bim in naïve CD19^+^CD27^–^ (Panel A) and memory CD19^+^CD27^+^ (Panel B) control B-cells unstimulated (left) and after activation with anti-BCR (middle) or anti-CD40 (right) in the presence or absence of IL-21 (respectively grey and white). Data are given as medians from 20 independent experiments (Wilcoxon test P-values: P<0.05*; P<0.01**; P<0.001***). MFI: median fluorescence intensity.

**Supplementary Figure 6. B-cells *in vitro* survival is differently influenced by IL-21 co-stimulation between healthy controls and CVID patients.**

Percentages of viable (Viability Dye-eFluor520 negative) naïve CD19^+^CD27^−^ (Panel A) and memory CD19^+^CD27^+^ (Panel B) B-cells unstimulated (circles) and after activation with IL-21 alone (squares), anti-BCR+IL-21 (rhombus) or anti-CD40+IL-21 (triangles) in controls (white), CVID (light grey) and apoptosis-prone CVID (dark grey) patients. Data are given as medians (Wilcoxon test P-values: P<0.01**; P<0.001***). HC: healthy controls (n=20); CVID: Common Variable Immunodeficiency patients (n=12); AP-CVID: apoptosis-prone CVID patients (n=7).

**Supplementary Figure 7. Distinct IL-21-induced levels of viable and apoptotic cells between control naïve and memory B-cells.**

Ratios of percentage of total Caspase-3-activated naïve CD19+CD27− (white bars) and memory CD19+CD27+ (grey bars) control B-cells (Panel A), unstimulated and after activation with IL-21 alone (left), anti-BCR+IL-21 (middle) or anti-CD40+IL-21 (right). Data are given as medians and 25-75th percentiles from 9 independent experiments (Mann–Whitney test P-values: P<0.05*; P<0.01**; P<0.001***). Percentages of viable, early-apoptotic, intermediate-apoptotic and late-apoptotic/necrotic cells in naïve CD19+CD27− (Panel B) and memory CD19+CD27+ (Panel C) control B-cells unstimulated (circles) and after activation with IL-21 alone (squares), anti-BCR+IL-21 (rhombus) or anti-CD40+IL-21 (triangles). Data are given as medians from 9 independent experiments (Wilcoxon test P-values: P<0.05*; P<0.01**).
